# Lifespan Pancreas Morphology for Control Versus Type 2 Diabetes Using AI on Largescale Clinical Imaging

**DOI:** 10.1002/ca.70077

**Published:** 2026-01-20

**Authors:** Lucas W. Remedios, Chloe Cho, Trent M. Schwartz, Dingjie Su, Gaurav Rudravaram, Chenyu Gao, Aravind R. Krishnan, Adam M. Saunders, Michael E. Kim, Shunxing Bao, Thomas A. Lasko, Alvin C. Powers, Bennett A. Landman, John Virostko

**Affiliations:** 1 Department of Computer Science, Vanderbilt University, Nashville, USA; 2 Department of Biomedical Engineering, Vanderbilt University, Nashville, USA; 3 Department of Electrical and Computer Engineering, Vanderbilt University, Nashville, USA; 4 Department of Biomedical Informatics, Vanderbilt University, Nashville, USA; 5 Department of Medicine, Division of Diabetes, Endocrinology, and Metabolism, Vanderbilt University Medical Center, Nashville, USA; 6 Department of Molecular Physiology and Biophysics, Vanderbilt University, Nashville, USA; 7 VA Tennessee Valley Healthcare System, Nashville, USA; 8 Department of Diagnostic Medicine, Dell Medical School, University of Texas at Austin, Austin, USA; 9 Dell Medical School, University of Texas at Austin, Livestrong Cancer Institutes, Austin, USA; 10 Department of Oncology, Dell Medical School, University of Texas at Austin, Austin, USA; 11 Oden Institute for Computational Engineering and Sciences, University of Texas at Austin, Austin, USA

**Keywords:** aging, CT, MRI, multimodal, pancreas, shape, volume

## Abstract

Understanding how pancreas size and shape change with normal aging is critical for establishing a baseline to detect deviations in type 2 diabetes and other pancreatic disease. We measure pancreas size and shape using morphological measurements from early development through aging (ages 0–90). Our goals are to (1) identify reliable clinical imaging modalities for artificial intelligence (AI) based pancreas measurement, (2) establish normative morphological aging trends, and (3) detect potential deviations in type 2 diabetes. We analyzed a clinically acquired dataset of 2533 patients imaged with abdominal computed tomography (CT) or magnetic resonance imaging (MRI). The patients did not have cancer, pancreas pathology, sepsis, or trauma. We resampled the scans to 3 mm isotropic resolution, segmented the pancreas using automated methods, and extracted 13 morphological pancreas features across the lifespan. First, we assessed pancreas volume trajectories in 1858 control patients across contrast CT, non-contrast CT, and MRI to determine which modalities provide consistent lifespan trends. Second, we characterized distributions of normative morphological patterns stratified by age group and sex. Third, we used covariate-adjusted generative additive models for location, scale, and shape (GAMLSS) regression to model pancreas morphology trends in 1350 patients matched for age, sex, and type 2 diabetes status to identify any deviations from normative aging associated with type 2 diabetes. We selected CT for the main analyses of this study, since the MRI appeared to yield different pancreas measurements than CT using our AI-based method on this dataset of clinically acquired scans. When adjusting for confounders, the aging trends for 10 of 13 morphological features were significantly different between patients with type 2 diabetes and non-diabetic controls (*p* < 0.05 after multiple comparisons corrections). Additionally, we characterized normative morphological aging trends of the pancreas across 13 morphological measurements. We provide lifespan trends demonstrating that the size and shape of the pancreas are altered in type 2 diabetes using 675 control patients and 675 diabetes patients. Moreover, our findings reinforce that the pancreas is smaller in type 2 diabetes. Additionally, we contribute a reference of lifespan pancreas morphology from a large cohort of non-diabetic control patients in a clinical setting.

## 1 ∣ Introduction

**FIGURE 1 ∣:**
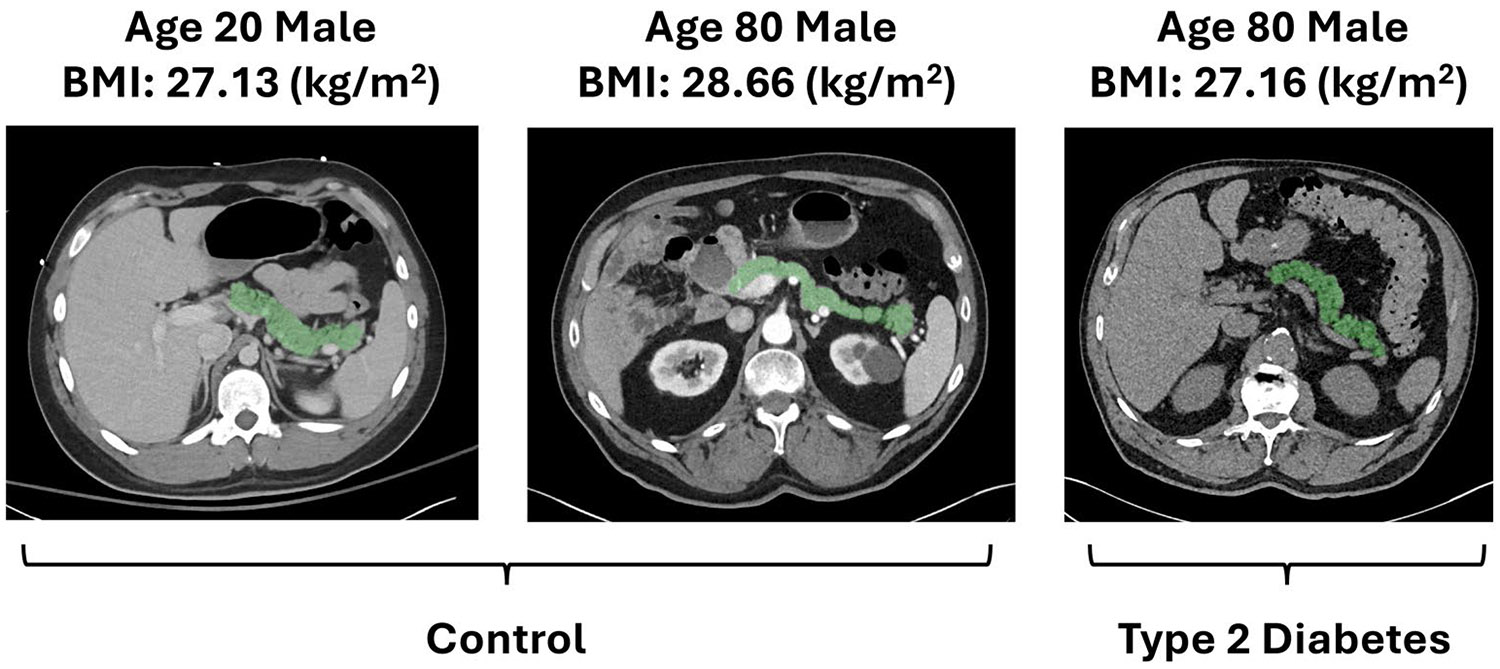
The pancreas undergoes structural changes with age, including atrophy and fat infiltration. While population-level pancreas volume and fat content have been examined across the aging process (Saisho et al. 2007), there remains a knowledge gap in understanding age-related changes in the pancreas across a broader set of morphological measurements. Moreover, type 2 diabetes may cause changes in pancreas morphology that differ from normal aging. The two scans on the left illustrate age-related appearance differences in non-diabetic patients but are not from the same patient. The rightmost scan shows the pancreas from an elderly patient with type 2 diabetes. Understanding pancreas variation in normal aging is critical for understanding differences in type 2 diabetes. Any potential differences in the aging trends of the pancreas in type 2 diabetes may not necessarily be linear or smooth.

Substantial efforts have been made to measure the morphology of the structural aging of organs, such as the brain, which helps distinguish between atrophy caused by normal aging and atrophy caused by disease (Statsenko et al. 2023). Defining an organ’s normative morphological aging allows investigation into how disease alters this trajectory, offering insight into the disease process. As with the brain, the pancreas also undergoes changes with age (Löhr et al. 2018) (Figure 1).

As the pancreas ages, it changes in size, shape, fat content, and duct structure, along with increasing fibrosis (Möller et al. 2023; Hastier et al. 1998; Saini et al. 2015; Glaser and Stienecker 2000; Kreel and Sandin 1973; Gupta et al. 2017). Separately from aging, pancreas morphology is influenced by sex and body composition (Dogan et al. 2021; Wang et al. 2021). Additionally, pancreatic morphological alterations have been extensively studied and reported in type 2 diabetes.

Pancreas volume is a critical measure of pancreas morphology. Nearly 20 years ago, Saisho et al. (2007) measured pancreas volume across the lifespan, where the pancreas was segmented (outlined by hand) from computed tomography (CT). Today, artificial intelligence (AI) enables faster, fully-automated segmentation of the pancreas (Dogan et al. 2021). Pancreas volume declines with age and is partially replaced by ectopic fat (Saisho et al. 2007; Löhr et al. 2018; Chantarojanasiri et al. 2015). In type 2 diabetes, the pancreas has been found to be smaller than control subjects, and has a serrated edge (Desouza et al. 2018; Macauley et al. 2015). In Saisho et al. (2007), the pancreas was determined to be smaller in subjects with type 2 diabetes in a large dataset matched for age, sex, and body mass index (BMI)—this finding aligned with previous studies (Fonseca et al. 1985; Alzaid et al. 1993; Gilbeau et al. 1992; Klöppel et al. 1985; Migdalis et al. 1991), but also differed from other studies that found no volume difference in type 2 diabetes from control subjects (Silva et al. 1993; Garcia et al. 2017). However, it is worth noting that the Silva et al. (1993) study was from ultrasound in the early 1990’s when precision was lower.

Beyond volume, deep characterization of pancreas morphology may lead to insights on disease states. The diameter of the pancreas has been identified as an important feature of pancreatic health (Desouza et al. 2018). Additionally, pancreas shape features have been extracted on magnetic resonance imaging (MRI) to study nearly 4000 subjects (Bagur et al. 2020).

**FIGURE 2 ∣:**
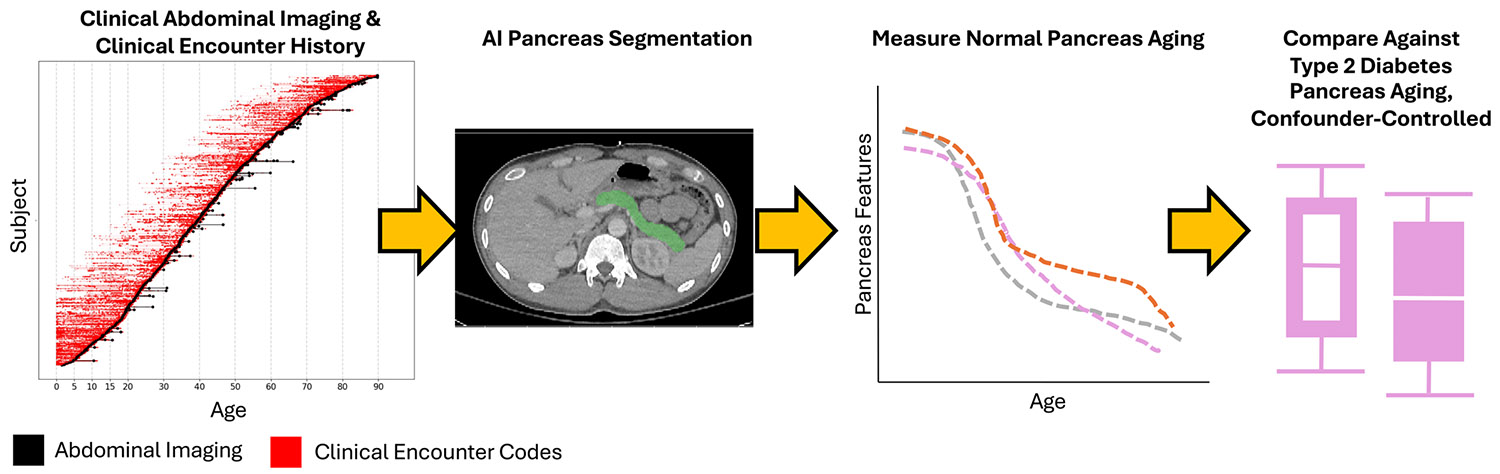
We leverage over 2500 clinical abdominal scans (CT or MRI) from control and type 2 diabetes patients. Using the AI tool TotalSegmentator (Wasserthal et al. 2023; Akinci D’Antonoli et al. 2025), we automatically segment the pancreas. We then use PyRadiomics (van Griethuysen et al. 2017) to extract 13 morphological measurements across the lifespan to assess whether pancreas aging differs in type 2 diabetes. We control for age, sex, and weight effects through both matching and modeling.

In this work, we measure the pancreas from a large clinical dataset via AI-driven pancreas segmentation (Figure 2). We comment on the consistency of clinical CT and MRI for volume measurements and present lifespan trends on normative aging with 13 morphological features of the pancreas. Further, we test for differences in structural aging between control and type 2 diabetes in a dataset matched on sex and age spanning ages 20 to 90. Rather than correcting for body size through division, we model aging trends while accounting for the covariates (age, sex, weight, and diabetes status).

## 2 ∣ Methods

### 2.1 ∣ Data

Our dataset was retrieved in deidentified form from site and IRB redacted for anonymization. We extracted features from an initial pool of 32,894 clinically acquired medical images. Because these data were obtained for routine clinical care rather than under a standardized research protocol, only a subset met our stringent quality assurance and inclusion criteria detailed below. After full processing and cohort selection, there were 2533 patients, each with one abdominal scan (CT or MRI), and each with a single diabetes label, either control or type 2 diabetes. For compatibility with downstream processing, the images were converted from DICOM to NIfTI format via dcm2niix (Li et al. 2016).

### 2.2 ∣ AI Segmentation & Feature Extraction

The scans were converted into LAS orientation, cropped between the L5 and T7 vertebrae (TotalSegmentator (Wasserthal et al. 2022) vertebrae segmentation), and resampled to 3 mm isotropic resolution. The pancreas and other abdominal organs were segmented with TotalSegmentator (Wasserthal et al. 2022; D’Antonoli et al. 2024) version 2.8, using the CT model for CT scans, and the MRI model for MRI scans. Thirteen morphological features were extracted from the binary NIfTI pancreas segmentations using PyRadiomics (van Griethuysen et al. 2017) version 3.1. The features were: volume, surface area, surface area to volume ratio, elongation, flatness, sphericity, major axis length, minor axis length, least axis length, maximum 3D diameter, maximum 2D diameter column, maximum 2D diameter row, and maximum 2D diameter slice.

### 2.3 ∣ Quality Control of Medical Images & Segmentations

To ensure data quality, all images and organ segmentations were manually inspected with a high-throughput visualization tool (Kim et al. 2024). In some cases, the conversion from DICOM to NIfTI format failed and corrupted image metadata. In these DICOM to NIfTI failure cases, the images were corrupted and exhibited geometric warping to varying degrees across the axial, sagittal, and coronal axes. The resulting warping produced distorted representations of pancreatic and abdominal anatomy that did not reflect true anatomical structure and would confound quantitative morphometric measurements if retained. Additionally, undesirable cases contained organ segmentations with more than one contiguous volume or that touched the edge of the image volume, indicating the organ extended beyond the field-of-view. These scans were excluded, with the warping/corruption identified via out-of-distribution examples of the ratio of the faces on a bounding box of the liver segmentation.

A subsequent manual inspection of the images and segmentations revealed that scans corrupted by geometric warping persisted in the dataset following these initial exclusion steps. In imaging sessions containing multiple acquisitions, such corruption affected only a subset of scans, necessitating a procedure to identify the uncorrupted acquisition within each session. To support this technical quality-control step, variability in organ volumes across repeated scans within the same session was evaluated across multiple abdominal organs (pancreas, liver, spleen, left kidney, and right kidney). Smooth polynomial reference trends across age were used solely as a technical envelope to identify implausible volume distortions consistent with the geometric warping artifacts, rather than as a model of biological variation. Within each session, scans were ranked based on their deviation from these reference trends with equal weighting across the five organs, and the scan least affected by such distortion was retained, while scans exhibiting warping artifacts/corruption were excluded. This approach involved a tradeoff between removing corrupted data and preserving biological variability; however, it was applied exclusively to address imaging artifacts and not to select scans based on biological characteristics or downstream analysis outcomes. All retained scans were subsequently verified by manual inspection, and all warped scans were excluded from the dataset.

### 2.4 ∣ Diabetes Label Assignment

To assign diabetes labels to the patients, we used their health records up to 1 year after the selected scan. International classification of disease (ICD) codes and derived PhecodeX (Shuey et al. 2023) were used to determine control patients as those without any diabetes events, and patients with type 2 diabetes as those with at least one type 2 diabetes event and no type 1 diabetes events. Where available, A1C measurements were used to validate that the control patients did not have diabetes. Using diagnosis information from ICD and PhecodeX, as well as procedure information from their current procedural terminology (CPT) and procedure wide association (ProWAS) codes, we excluded patients with cancer, pancreas pathology, and sepsis (Chaganti et al. 2017; Kerley et al. 2022). Pancreas pathology was identified by filtering ICD and PhecodeX records for the word segment “panc.” We also excluded imaging associated with trauma events to avoid scans with acute structural and functional alterations (e.g., hemorrhage, edema).

### 2.5 ∣ Automatic Contrast Phase Labeling of CT

CT scans were automatically labeled according to contrast phase using TotalSegmentator’s contrast phase prediction tool (Wasserthal et al. 2022). These labels were inspected and determined to be accurate for binary contrast labeling (contrast CT or non-contrast CT). The final binary phase classification was manually assured for each CT through visual inspection of the scans using AutoQA (Kim et al. 2024).

### 2.6 ∣ Pancreas Measurement Consistency Across CT & MRI

Before analyzing anatomical trends, we needed to select which imaging modalities (CT or MRI) in the clinical dataset consistently measured the pancreas. We chose to use pancreas volume, pancreas volume index (volume divided by patient weight), and BMI of healthy controls to assess this. Based on observation of lifespan polynomials fit with 95% confidence intervals, we selected the subset of modalities to use in subsequent analyses.

### 2.7 ∣ Lifespan Pancreas Morphology in the Clinic by Sex

On our selected modalities, we created a lifespan reference of normative pancreas morphology by creating boxplots to visualize the distributions of each uncorrected pancreas morphological feature by sex and age group as in Saisho et al. (2007). These data were separated by sex but were not matched.

### 2.8 ∣ Modeling Pancreas Aging With and Without Diabetes

We modeled each pancreas feature across the adult lifespan (ages 20–90) using a single generalized additive model for location, scale, and shape (GAMLSS (Stasinopoulos and Rigby 2007)), jointly incorporating both male and female, as well as individuals with and without type 2 diabetes. The location (μ) was modeled as:

(1) $$\mu =\beta_{0}+f(\text{age})+\beta_{1}\;\text{diabetes}+\beta_{2}\;\text{sex}+\beta_{3}\;\text{weight}$$

Here $\beta_{0}$ is the intercept, and $\beta_{1}$, $\beta_{2}$, and $\beta_{3}$ are coefficients for type 2 diabetes status, sex, and weight respectively. The term (age) represents a smooth, nonlinear function of age, implemented using penalized B-splines. We selected this GAMLSS model to flexibly capture the nonlinear nature of biological aging. In contrast, diabetes, sex, and weight were modeled linearly under the assumption that they introduce global shifts. The same covariates were included in the model for the scale (σ), and we used the Box-Cox Cole and Green (BCCG) distribution to handle skewness in the feature distributions. Although GAMLSS does not include an explicit residual term like in traditional linear models, the progression-related variability (i.e., error) is captured through the distribution parameters. The GAMLSS approach enables learning distributions, rather than just the mean, as a function of the input variables.

Although one model was fit per pancreas feature, we visualized the fitted curves separately for male and female to highlight sex-specific differences. For each sex, weight was fixed to the average weight across all patients of that sex (both type 2 diabetes and control), and we conditioned the visualization on type 2 diabetes status to isolate its impact on pancreas aging. Because we modeled type 2 diabetes diagnosis as a linear relationship, we obtained a single *p*-value for the differences between the type 2 diabetes and control groups. These *p*-values were corrected using the Benjamini– Hochberg False Discovery Rate (FDR) to account for the 13 features tested. After FDR correction, *p* < 0.05 was considered significant.

### 2.9 ∣ Use of Generative AI in Manuscript

Generative AI (ChatGPT4o) was employed to assist with drafting and content refinement throughout this research. All core intellectual content and insights originated from the authors’ independent scholarly work. All AI-generated material passed through careful author review and revision to ensure alignment with the study’s objectives and to maintain academic rigor.

## 3 ∣ Results

### 3.1 ∣ Comparison of Clinical Imaging Modality for Measuring the Pancreas

**FIGURE 3 ∣:**
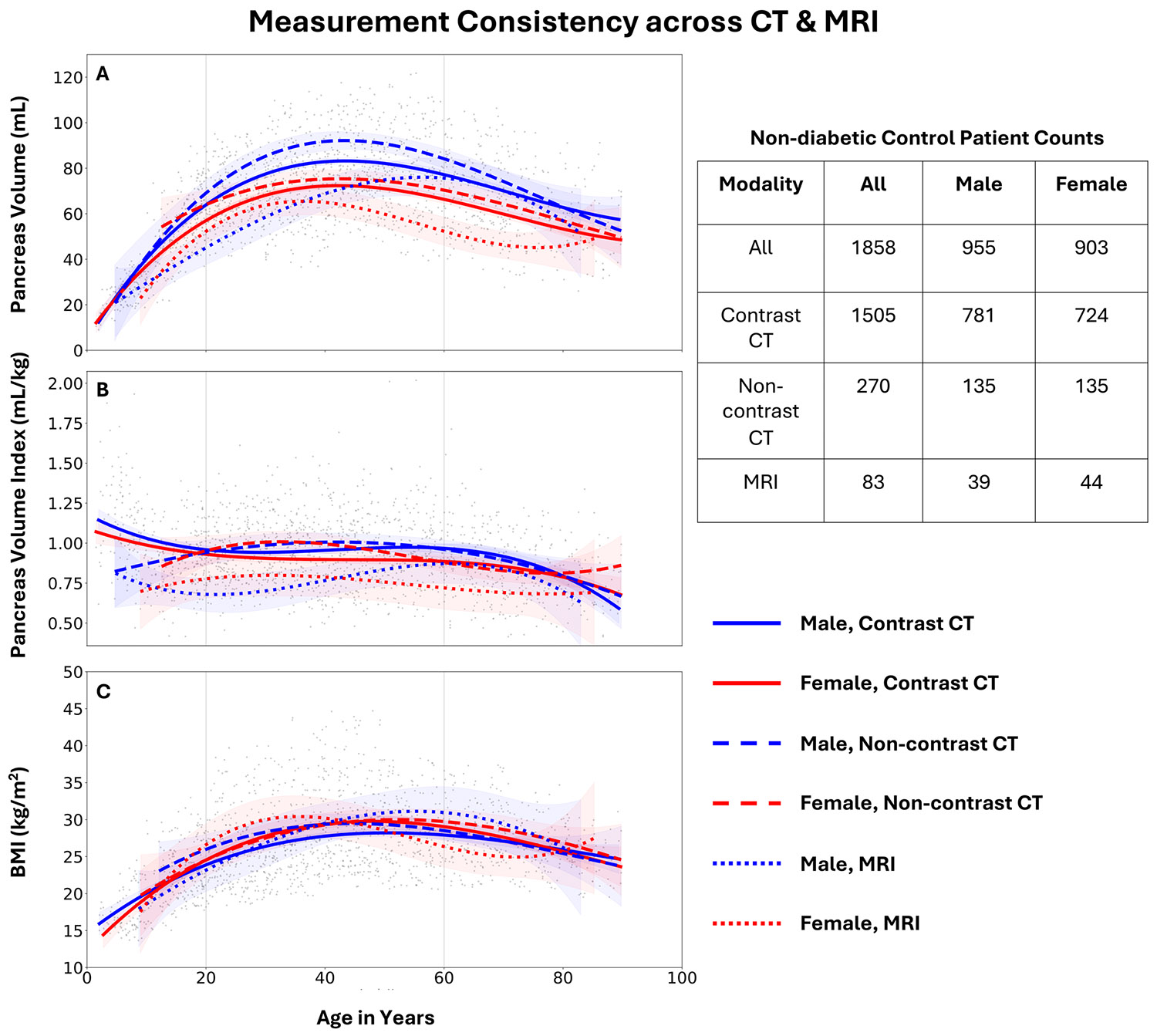
We investigate measurement consistency between CT and MRI using lifespan polynomials. With our automatic AI approach, MRI appears to measure a smaller pancreas volume than CT for most age groups (A). The observable difference between the MRI and CT trends becomes more evident when correcting for body size by dividing pancreas volume by patient weight (B). BMI is comparable across the modalities, which further implies that observed reduced pancreas measurements in MRI are likely not caused by smaller body size (C). These trends are from non-diabetic control patients. Given the observed discrepancy in automated pancreas volume measures between MRI and CT, we cautiously exclude the smaller cohort of MRI measurements from further analyses and proceed with CT measurements.

In Figure 3, we observed for agreement between contrast CT, non-contrast CT, and MRI for measuring the pancreas via lifespan trends (polynomials). We selected pancreas volume as the main feature, since population-level reference ranges are provided by Saisho et al. (2007). We used an unmatched subset of the dataset consisting of all 1858 control patients. We observed that MRI tended to yield smaller pancreas measurements than CT. To account for differences in weight distributions, we also assessed the pancreas volume index (pancreas volume divided by patient weight), where the pattern was still observed. We additionally checked the distribution of BMI across modalities, which was comparable. Based on this observed incongruency between image modalities, we cautiously excluded automated pancreas segmentation derived from MRI from our subsequent analyses and instead focused on the larger group of CT images.

### 3.2 ∣ Lifespan Pancreas Morphology Shows Aging Trends

**FIGURE 4 ∣:**
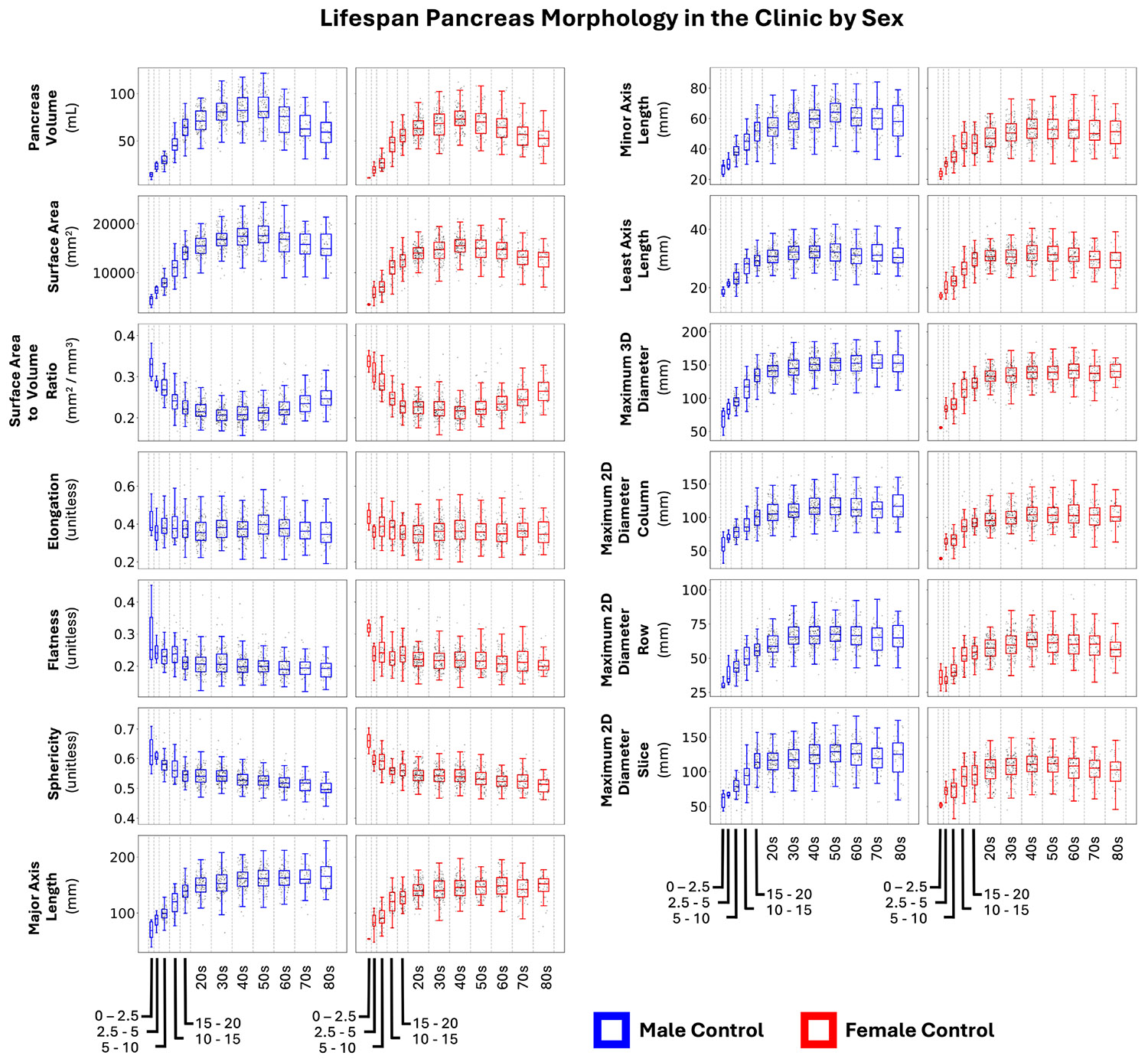
From a population of 1775 non-diabetic control patients with CT scans, we illustrate how pancreas size and shape change with age and sex across 13 morphological measurements. We reproduce findings from Saisho et al. on pancreas volume (Saisho et al. 2007). These distributions visualize population spread of pancreas measurements across age groups.

In Figure 4, we present uncorrected lifespan morphology of the pancreas in an unmatched dataset of 1775 nondiabetic control patients with CT imaging of the pancreas. Our results reproduce the population volume trends from Saisho et al. (2007) Across size metrics (axis lengths and diameters), the female pancreas is generally smaller in adulthood.

### 3.3 ∣ Matched Dataset for Assessing Control Versus Type 2 Diabetes

**FIGURE 5 ∣:**
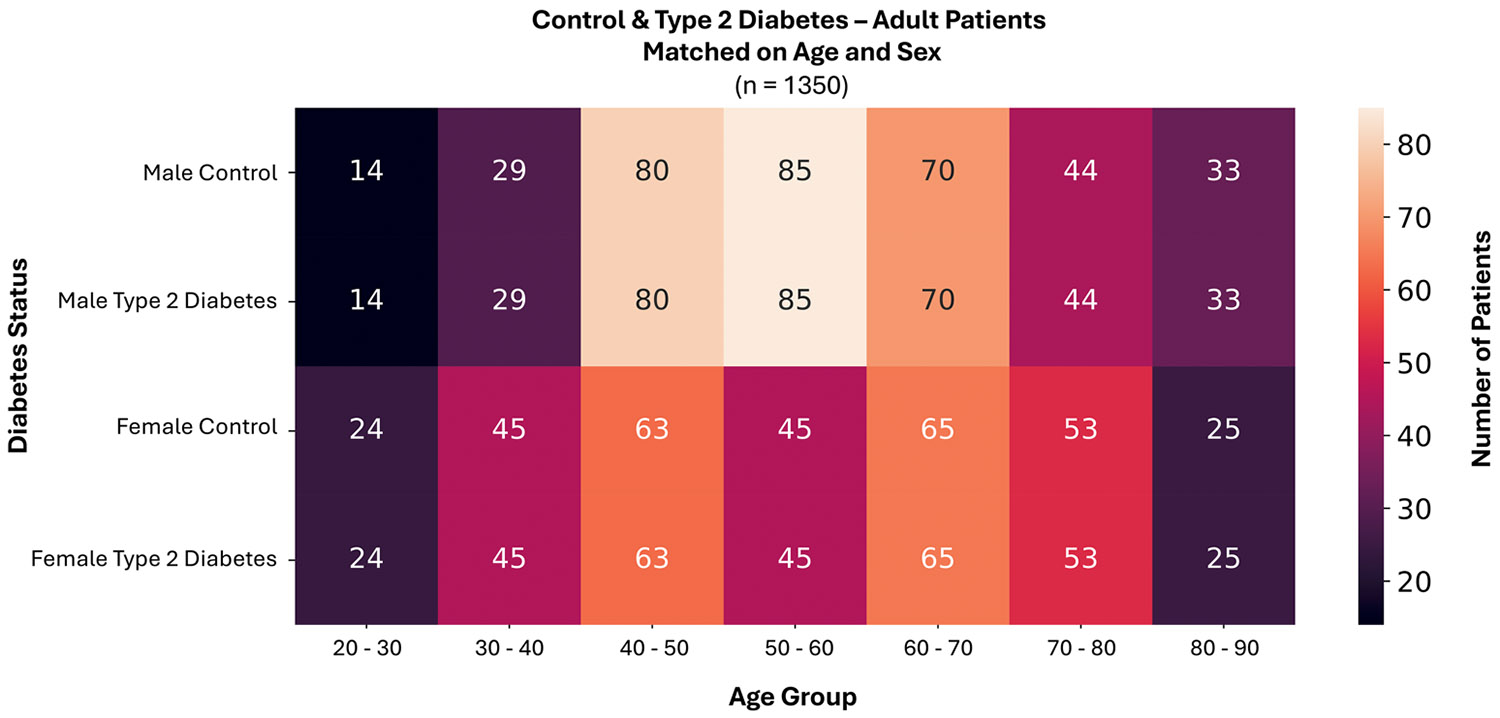
We used a matched subset of the data with 1350 patients to mitigate the confounding effects of sex and age before assessing whether pancreas morphology differs in type 2 diabetes. Body size was not matched but was addressed later through modeling. Each cell provides the number of patients, colored by the color bar, in each diabetes/sex and age group.

In Figure 5, we show the distribution of matched patients by age, sex, and diabetes status. These 1350 patients allow a large sample size for measuring potential adulthood pancreas aging differences in type 2 diabetes, with most of the data being available from age 40 to 70.

### 3.4 ∣ Shift in Diabetes

**FIGURE 6 ∣:**
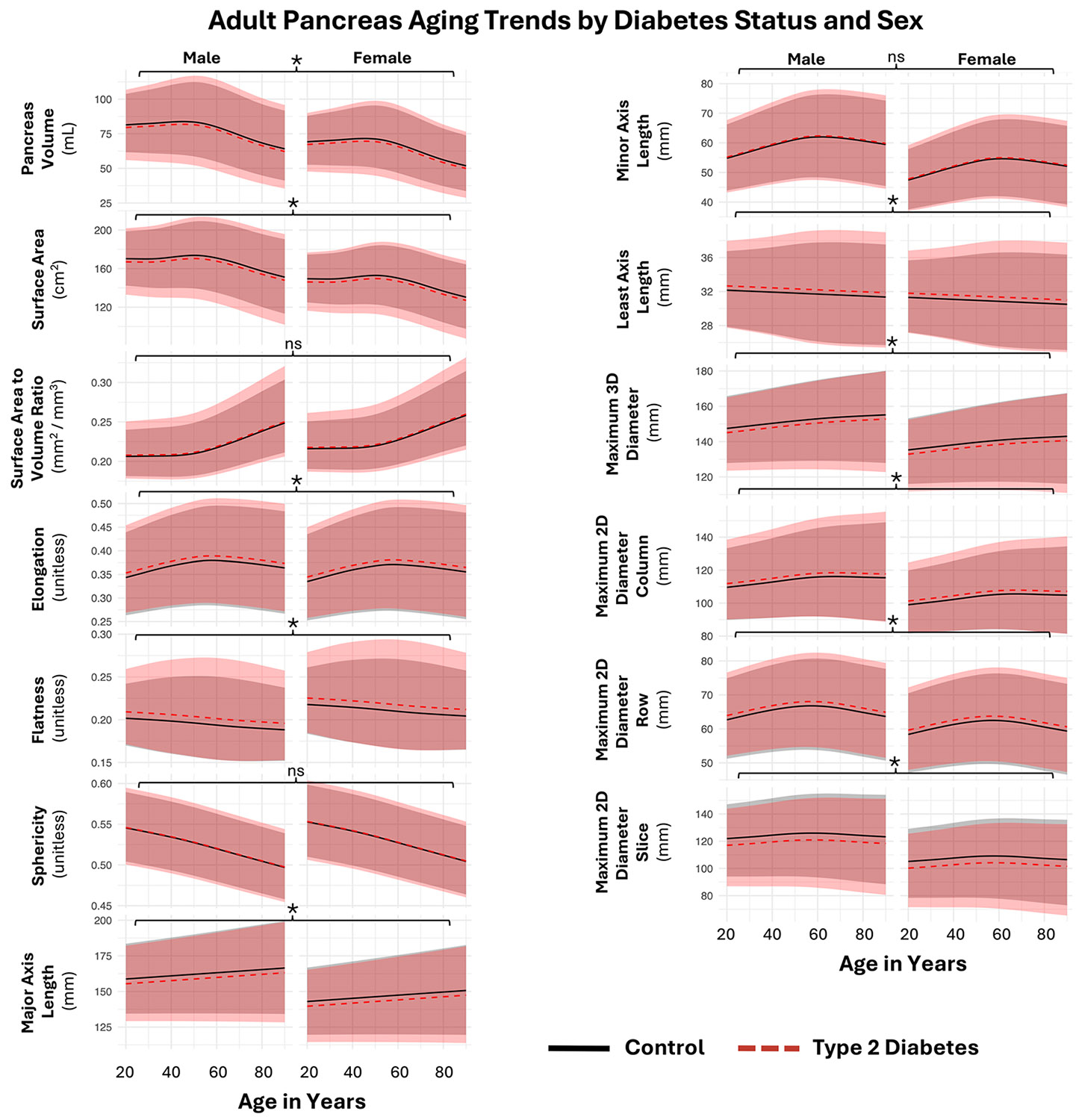
In type 2 diabetes, the pancreas is smaller and has an altered shape with our GAMLSS regression that models diabetes diagnosis with a linear term. Across these 13 morphological measurements, 10 of the aging trends were significantly different in diabetes, with *p* < 0.05 after multiple comparisons correction denoted with *. In these plots, we show curves that represent the 50th percentile (median) of the GAMLSS model-learned distributions, with the range from the 5th to 95th centiles shaded, holding other covariates constant. While we detect statistical significance via the linear type 2 diabetes parameter across most of the metrics, the distributions from the patients with type 2 diabetes are similar to the nondiabetic controls.

**FIGURE 7 ∣:**
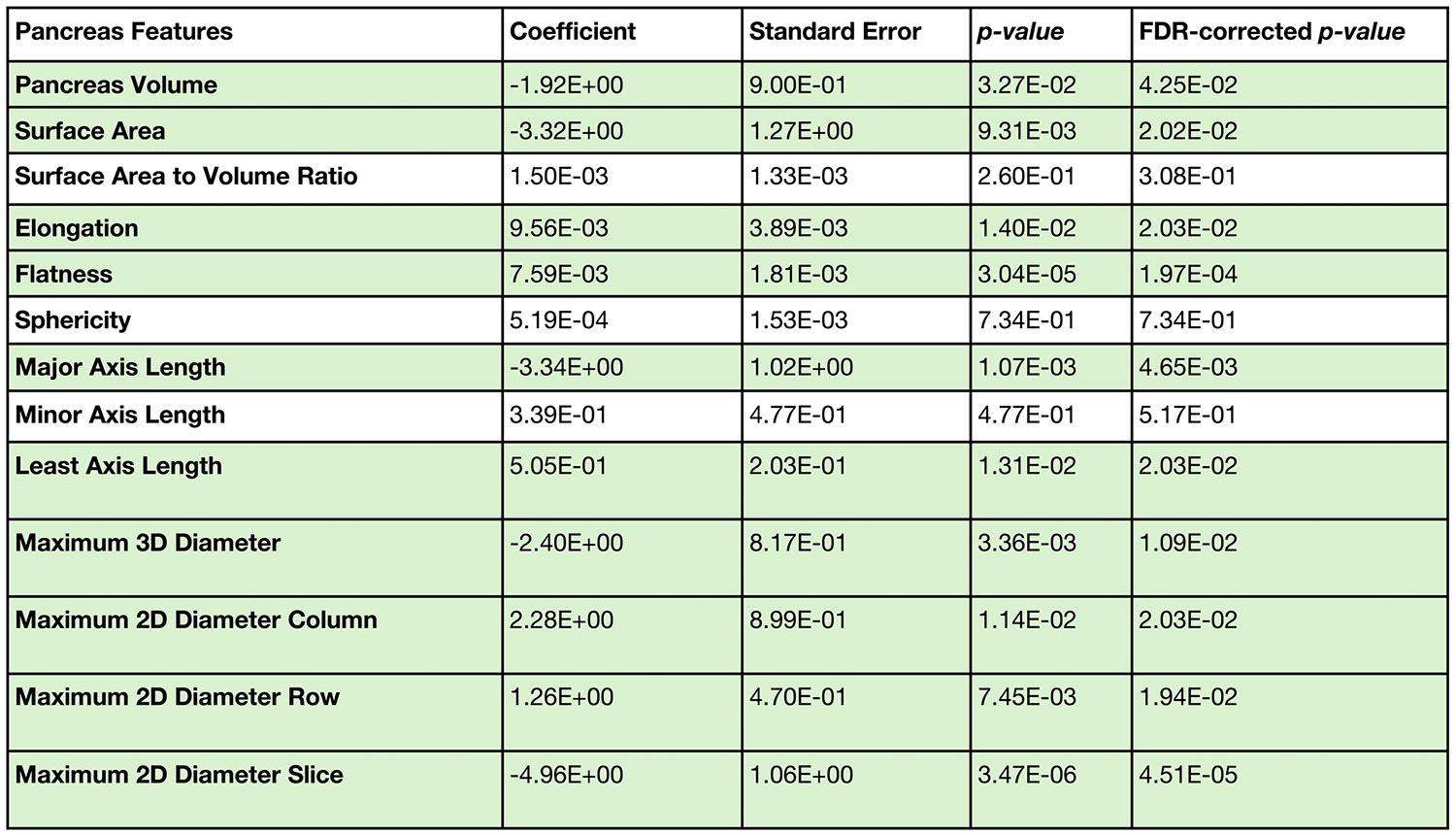
We complement the curves from Figure 6 with the numerical results for the type 2 diabetes linear coefficient from the μ of the learned distributions from the GAMLSS model. Statistical significance after FDR correction is denoted in green.

In Figure 6, we show that when accounting for covariates, 10 of 13 morphological measurements of the pancreas were significantly different (*p* < 0.05 after multiple comparisons corrections on the linear type 2 diabetes parameter). On the statistically significant features, there were small shifts between the type 2 diabetes and control curves, indicating that type 2 diabetes is associated with measurable changes in pancreas morphology throughout adulthood. These differences persisted even after both matching and modeling the data to account for age, sex, and body weight using GAMLSS. Significant differences were observed across most of the anatomical measurements, including pancreas volume, surface area, surface-to-volume ratio, elongation, flatness, sphericity, and multiple axis and diameter-based measurements. We also observed sex-specific trends, with shifts between male and female trajectories. The findings in Figure 6 support that the type 2 diabetes pancreas is smaller than control and has altered morphology throughout aging in adulthood, however there is a large amount of overlap in the distributions between type 2 diabetes and control. In Table 1, we complement Figure 6 by reporting decade-level reference values for pancreatic measurements derived from the same GAMLSS model used in Figure 6. For each measurement, the GAMLSS model estimated the median (50th percentile) at each integer age from 20 to 90 years. The decade-level values in Table 1 were obtained by taking the median of these age-specific model-estimated medians within each decade. In Figure 7, we present complementary numerical details for the type 2 diabetes coefficient from the μ of the GAMLSS model.

## 4 ∣ Discussion

In this study, we characterized age-related changes in pancreas size and shape using a large, clinically acquired dataset and AI-based segmentation across CT and MRI modalities. We found that pancreas morphology ages differently in type 2 diabetes across numerous measures of size and shape. Our modality comparison led to observed differences between MRI and CT in AI-based pancreas measurement, emphasizing the need for modality-specific baselines.

Measuring the pancreas from medical imaging is commonly performed on both CT and MRI in research settings. In clinical populations, CT is more prevalent; however, MRI is commonly performed in research studies to avoid radiation exposure (Virostko et al. 2021). In datasets of clinically acquired abdominal CT scans, images reflect real-world variability in image quality and acquisition protocols, which may influence pancreas appearance and measurement. Several previous studies have inspected the pancreas from CT and/or MRI (Syed et al. 2012; Caglar et al. 2012; Kipp et al. 2019; Zhou et al. 2025; Sato et al. 2012; Le Goallec et al. 2022; Remedios et al. 2025).

We observed that, within this clinically acquired dataset, AI-based pancreas volume measurements derived from MRI were systematically smaller than those derived from contrast and non-contrast CT. However, our AI-based measurements on CT reproduced previously reported lifespan trends from manual pancreas volumetry (e.g., Saisho et al. 2007), suggesting that the observed discrepancy in pancreas size between CT and MRI was specific to the application of our automated segmentation approach across modalities rather than to biological differences. Consistent with this observation, manual quality assurance identified a higher frequency of segmentation failures on MRI than on CT. This likely reflects challenges associated with the generalization of pretrained AI-based models on heterogeneous routine clinical MRI acquisitions without dataset-specific adaptation or harmonization.

Prior CT- and MRI-based studies have generally reported reduced pancreatic volume in individuals with type 2 diabetes. Saisho et al. (2007), for example, matched on sex, age, and BMI and found that the pancreas of people with type 2 diabetes was smaller than control. Our findings agree with the evidence pointing toward a smaller pancreas in type 2 diabetes. Moreover, we demonstrate that the shape of the pancreas is also altered in type 2 diabetes across several morphological measurements. Importantly, recent work has demonstrated that substantial weight loss in individuals with type 2 diabetes can return pancreas size and surface irregularity to normal (Al-Mrabeh et al. 2020). While an additional investigation of fat infiltration across the lifespan could prove insightful, the vast majority of our CT had contrast, which renders fat estimation from Hounsfield units inaccurate.

To compare subjects with different body sizes, studies often divide pancreas volume by body surface area (BSA), weight, or BMI—an approach sometimes used to assess differences between individuals with and without diabetes (Goda et al. 2001; Al-Mrabeh et al. 2016; Cai et al. 2025; Wright et al. 2022; Pollé et al. 2023; Vesterhus et al. 2008; Almeida et al. 2018; Fortson et al. 2024; Kawaji et al. 2021; Virostko et al. 2021). This approach is supported by demonstrations that there are correlations between pancreas volume and BSA, weight, and BMI (Macauley et al. 2015; Caglar et al. 2014; McCleary et al. 2020; Sequeira et al. 2022). In our comparison between patients with type 2 diabetes and non-diabetic controls, we accounted for body size variability by including weight as a covariate.

This work is limited in that we consider contrast in CT as a binary flag. In reality, contrast cycles through phases which impact the way the pancreas appears on imaging. Variability in contrast phase may influence pancreas segmentation via TotalSegmentator and may influence resulting measurements of the pancreas.

To advance our understanding of pancreas aging, future work should focus on quantifying changes in pancreatic size and shape using higher-resolution medical imaging to enable finer structural analysis. In parallel, evaluating how anatomical aging patterns vary across clinical sites and populations will help establish robust baselines. In this study, we focused on CT scans, since automated pancreas measurements differed between CT and MRI in this dataset and the available CT cohort was substantially larger. MRI-based methods can likewise provide precise anatomical information when applied under standardized acquisition protocols in controlled research settings; however, extending such AI-based analyses to large-scale collections of routine clinical MRI remains an active area of methodological development due to variability in acquisition parameters.

## 5 ∣ Conclusions

We find that in patients with type 2 diabetes, the pancreas is significantly smaller and different in shape than non-diabetic controls via a linear parameter for type 2 diabetes in the GAMLSS model. We provide reference trends for how control and type 2 diabetes pancreas morphology changes across the lifespan, as imaged in the hospital. The observed differences in AI-based pancreas measurements between clinically acquired CT and clinically acquired MRI in this large-scale dataset highlight that for cross-modality studies, it is essential to use robust image harmonization to ensure reliable pancreas measurements across imaging types. Without image harmonization, findings in one imaging modality may be difficult to interpret in the context of another.

## Figures and Tables

**TABLE 1 ∣:** Complementary to Figure 6, we present decade-level pancreatic reference values derived from the same GAMLSS model.

Model-estimated measurements	Sex	Diabetes status	20–29	30–39	40–49	50–59	60–69	70–79	80–89
Volume (mL)	M	Control	81.898	82.857	83.722	82.344	77.391	71.362	66.338
	M	T2DM	79.975	80.934	81.798	80.420	75.467	69.438	64.415
	F	Control	69.672	70.631	71.496	70.117	65.165	59.136	54.112
	F	T2DM	67.748	68.708	69.572	68.194	63.241	57.212	52.189
Surface area (cm^2^)	M	Control	170.119	170.479	173.003	173.143	168.472	161.443	154.516
	M	T2DM	166.800	167.160	169.685	169.824	165.154	158.125	151.197
	F	Control	149.305	149.665	152.190	152.329	147.659	140.629	133.702
	F	T2DM	145.986	146.347	148.871	149.011	144.340	137.311	130.384
Surface area to volume ratio (mm^2^/mm^3^)	M	Control	0.207	0.207	0.208	0.213	0.222	0.232	0.243
	M	T2DM	0.208	0.208	0.209	0.214	0.223	0.234	0.245
	F	Control	0.216	0.217	0.218	0.222	0.231	0.242	0.253
	F	T2DM	0.218	0.218	0.219	0.224	0.233	0.243	0.254
Elongation (unitless)	M	Control	0.349	0.362	0.372	0.379	0.378	0.374	0.368
	M	T2DM	0.359	0.371	0.382	0.389	0.388	0.383	0.377
	F	Control	0.341	0.353	0.364	0.371	0.370	0.365	0.359
	F	T2DM	0.350	0.363	0.373	0.380	0.379	0.375	0.369
Flatness (unitless)	M	Control	0.201	0.199	0.197	0.195	0.193	0.191	0.189
	M	T2DM	0.209	0.207	0.205	0.203	0.200	0.198	0.197
	F	Control	0.217	0.215	0.214	0.211	0.209	0.207	0.205
	F	T2DM	0.225	0.223	0.221	0.219	0.216	0.214	0.213
Sphericity (unitless)	M	Control	0.543	0.537	0.531	0.524	0.516	0.509	0.501
	M	T2DM	0.544	0.538	0.532	0.525	0.517	0.509	0.502
	F	Control	0.550	0.545	0.539	0.532	0.524	0.516	0.509
	F	T2DM	0.551	0.545	0.539	0.532	0.524	0.517	0.509
Major axis length (mm)	M	Control	159.243	160.349	161.456	162.563	163.668	164.772	165.876
	M	T2DM	155.901	157.008	158.115	159.221	160.327	161.431	162.535
	F	Control	143.469	144.576	145.682	146.789	147.894	148.999	150.102
	F	T2DM	140.128	141.235	142.341	143.448	144.553	145.657	146.761
Minor axis length (mm)	M	Control	55.779	57.998	60.072	61.676	61.944	61.250	60.132
	M	T2DM	56.118	58.337	60.411	62.016	62.283	61.589	60.471
	F	Control	48.416	50.636	52.709	54.314	54.581	53.887	52.770
	F	T2DM	48.756	50.975	53.048	54.653	54.920	54.226	53.109
Least axis length (mm)	M	Control	32.114	31.999	31.884	31.768	31.653	31.538	31.422
	M	T2DM	32.619	32.504	32.388	32.273	32.158	32.042	31.927
	F	Control	31.258	31.143	31.027	30.912	30.796	30.681	30.566
	F	T2DM	31.763	31.647	31.532	31.417	31.301	31.186	31.071
Maximum 3D diameter (mm)	M	Control	148.115	149.556	150.995	152.305	153.403	154.203	154.836
	M	T2DM	145.716	147.157	148.596	149.906	151.004	151.804	152.437
	F	Control	135.953	137.394	138.833	140.143	141.241	142.040	142.673
	F	T2DM	133.554	134.995	136.434	137.744	138.842	139.642	140.274
Maximum 2 D diameter column (mm)	M	Control	110.291	111.864	113.643	115.292	116.114	115.982	115.574
	M	T2DM	112.570	114.143	115.922	117.571	118.394	118.261	117.853
	F	Control	99.717	101.290	103.069	104.718	105.541	105.408	105.000
	F	T2DM	101.996	103.570	105.348	106.997	107.820	107.688	107.279
Maximum 2D diameter row (mm)	M	Control	63.382	64.881	66.077	66.749	66.532	65.532	64.306
	M	T2DM	64.642	66.140	67.336	68.008	67.792	66.791	65.565
	F	Control	59.087	60.585	61.781	62.453	62.236	61.236	60.010
	F	T2DM	60.346	61.845	63.041	63.713	63.496	62.495	61.270
Maximum 2D diameter slice (mm)	M	Control	122.471	123.683	125.076	125.902	125.729	124.765	123.758
	M	T2DM	117.515	118.727	120.120	120.946	120.774	119.810	118.803
	F	Control	105.678	106.890	108.283	109.109	108.937	107.973	106.966
	F	T2DM	100.722	101.934	103.328	104.154	103.982	103.019	102.014

*Note:* Values represent the median obtained by aggregating age-specific model-estimated medians (50th percentiles) within each decade.

## Data Availability

Research data are not shared.
